# Population dynamics and antigenic drift of *Bordetella pertussis* following whole cell vaccine replacement, Barcelona, Spain, 1986–2015

**DOI:** 10.1080/22221751.2019.1694395

**Published:** 2019-11-26

**Authors:** Alba Mir-Cros, Albert Moreno-Mingorance, M. Teresa Martín-Gómez, Gema Codina, Thais Cornejo-Sánchez, Mireia Rajadell, Diego Van Esso, Carlos Rodrigo, Magda Campins, Mireia Jané, Tomàs Pumarola, Anna Fàbrega, Juan José González-López

**Affiliations:** a Department of Clinical Microbiology, Hospital Universitari Vall d'Hebron, Barcelona, Spain; b Department of Microbiology and Genetics, Universitat Autònoma de Barcelona, Barcelona, Spain; c Primary Care Health Centre Service 'Muntanya', Catalan Institute of Health, Barcelona, Spain; d Department of Paediatrics, Hospital Universitari Vall d'Hebron, Barcelona, Spain; e Department of Preventive Medicine and Epidemiology, Hospital Universitari Vall d'Hebron, Barcelona, Spain; f Public Health Agency of Catalonia, Barcelona, Spain; g Faculty of Health Sciences, University of Vic – Central University of Catalonia (UVic-UCC), Manresa, Spain

**Keywords:** Whooping cough, PFGE, MLVA, antigenic variants, pertussis vaccine

## Abstract

Among the factors associated with the resurgence of whooping cough, special emphasis has been given to pathogen adaptation after the introduction of the acellular vaccine (ACV). To assess the impact of the vaccine transition strategy from whole-cell vaccine (WCV) to ACV on population dynamics of *Bordetella pertussis* in Barcelona (Spain), we studied 339 isolates collected from 1986 to 2015 by PFGE and multi-locus variable-number tandem repeat analysis (MLVA). Additionally, allelic variants for the pertussis toxin and its promoter, pertactin, type 3 fimbriae and fimbrial serotyping were assessed to determine its antigenic drift. A shift was observed in the *B. pertussis* population as well as in its antigenic profile concurrently with the introduction of ACV in Barcelona. Four out of the five most prevalent PFGE profiles were replaced by new profiles following the ACV introduction. MLVA type 27 was the dominant genotype, and its frequency increased from 25% to 79.3% after WCV replacement. Antigen typing demonstrated the emergence of *prn2*, *ptxP3*, *fim3-2* and a shift from the fimbriae 3 to the fimbriae 2 serotypes after the ACV introduction. Our findings support the presence of population and antigenic dynamic changes in *B. pertussis* likely driven by the introduction of ACV.

## Introduction

Whooping cough, or pertussis, is an acute human upper respiratory tract infection caused by *Bordetella pertussis*, a highly communicable airborne Gram-negative coccobacillus. The pertussis immunization programme was first introduced in Spain in 1965, with the administration of a whole cell vaccine (WCV). Later, in 1998 an acellular vaccine (ACV) progressively replaced the WCV vaccination programme with the aim of improving vaccine safety. Finally, since 2005 ACV is the only vaccine administered to the Spanish population [1]. In Catalonia, the vaccination programme against pertussis follows the same programme (Table 1).

**Table 1.** Pertussis vaccination programme used in Catalonia (Spain) since 1965.

	Primary doses			Booster doses		
Year	Vaccine type	Schedule	Pertussis components	Vaccine type	Schedule	Pertussis components
1965	DTPw	2 doses between 3 months and 3 years	Inactivated whole cell	NA	No booster	NA
1967	DTPw	3 doses between 3 months and 3 years	Inactivated whole cell	NA	No booster	NA
1975	DTPw	3, 5, 7 months	Inactivated whole cell	NA	No booster	NA
1996	DTPw	2–3, 4–5, 6–7 months	Inactivated whole cell	DTPw	15–18 months	Inactivated whole cell
1998–1999	DTPw	2, 4, 6 months	Inactivated whole cell	DTPa	18 months 4–6 years	PT, FHA, PRN
2000	DTPw/DTPa	2, 4, 6 months	Inactivated whole cell or PT, FHA, PRN	DTPa	18 months 4–6 years	PT, FHA, PRN
2002	DTPa	2, 4, 6 months	PT, FHA, PRN	DTPa	18 months 4–6 years	PT, FHA, PRN
2011	DTPa	2, 4, 6 months	PT, FHA, PRN	DTPa/dTpa	18 months (DTPa), 4–6 years (dTpa)	PT, FHA, PRN or PT, FHA, PRN, FIM2, FIM3^a^
2014	DTPa	2, 4, 6 months	PT, FHA, PRN	DTPa/dTpa	18 months (DTPa), 6 years (dTpa)	PT, FHA, PRN or PT, FHA, PRN, FIM2, FIM3^a^
2014^b^	NA	NA	NA	dTpa	between 27 through 36 weeks of pregnancy	PT, FHA, PRN or PT, FHA, PRN, FIM2, FIM3

Notes: NA: not applicable; DTPw: diphtheria-tetanus-whole cell pertussis vaccine; DTPa: diphtheria-tetanus-acellular pertussis vaccine; dTpa: diphtheria-tetanus-accellular pertussis vaccine with reduced antigenic load of diphtheria, tetanus and pertussis; PT: pertussis toxin; FHA: filamentous haemagglutinin; PRN: pertactin; FIM2: type 2 fimbriae; FIM3: type 3 fimbriae. These data have been collected from Campins M, *et al* ([1] and personal communication).

^a^The composition containing pertussis fimbrial antigens is used in some cases for the fifth dose.

^b^Introduction of maternal pertussis vaccination.

Despite extensive vaccination campaigns and high immunization rates (86% for global primary vaccination in 2018), the incidence of whooping cough significantly reemerged during the first decade of the twenty-first century, not only in Spain and Europe but also worldwide. This unfavourable situation has led to pertussis becoming the leading vaccine-preventable disease in industrialized countries, and hence, a global public health problem [2–5].

Pertussis normally presents a classical cyclic pattern with epidemic waves occurring every 3–5 years interspersed with periods of a lower incidence rate. However, at present, the number of cases is higher than in previous decades [6]. The reemergence of pertussis in Spain is particularly well supported by recent epidemiological data. The incidences reported for the interepidemic years from 2001 to 2010 ranged from 0.7 to 1.9 cases/100,000 population. However, despite having a vaccination coverage of 96.5% in infants younger than 12 months, the incidence rates observed after the last two epidemic waves (2011 and 2015) remained high even during interepidemic periods (>5 cases/100,000 population). Of note was the increase reported during the last epidemic wave in 2015 which showed a maximum incidence of almost 18 cases/100,000 population [7]. The figures reported in Catalonia have followed a similar trend albeit with an even more pronounced progression, with an incidence of 49 cases/100,000 population in 2015 [8].

The resurgence of whooping cough may be related to increased awareness and improved diagnostic tools, although the special emphasis has been given to waning immunity, particularly associated with the introduction of ACV and pathogen adaptation [9,10]. ACV contains a combination of different antigens including pertussis toxin (PT), pertactin (PRN) and filamentous haemagglutinin (FHA), and in some vaccines, type 2 and type 3 fimbriae (FIM2 and FIM3) [2]. Nevertheless, the immunologic protection conferred by ACV is not as enduring as that previously observed for WCV [11]. The current allelomorphic profile of the ACV antigens includes *ptxA2/ptxA4*, *prn1/prn7, fhaB1, fim2-1* and *fim3-1* [12]. The naturally-driven antigenic divergence, together with the impact exerted by the ACV vaccine has likely played a key role in the selection of new antigenic variants, which is in keeping with the increased frequencies of the allelic variants *ptxA1*, *prn2*, and *fim3-2* seen in countries where ACV has been extensively used [2,4,13]. Another bacterial adaptation event associated with the resurgence of this pathogen is the selection of a new PT promoter type. In 2009 the new *ptxP3* variant was characterized in relation to increased toxin production levels and was shown to have rapidly replaced the previous predominant *ptxP1* allele [14,15].

The main objective of this study was to shed new light on the impact of the introduction of ACV on the evolution and adaptation of *B. pertussis* isolates over a 30-year period in Spain, particularly those collected in the metropolitan area of Barcelona before, during and after the WCV replacement by ACV. This characterization was achieved by molecular epidemiology analysis of clinical isolates together with a study of the vaccine antigen variants of the pathogen.

## Materials and methods

### Bacterial isolates and study period

A total of 339 non-duplicate *B. pertussis* clinical isolates were collected at the Hospital Vall d’Hebron (Barcelona, Spain). All the isolates were recovered from cultures of nasopharyngeal samples collected from patients diagnosed with pertussis, excluding isolates of the studies of contacts. The isolates were collected from patients with different vaccination status: vaccinated, unvaccinated and partially vaccinated (Table S1, Supplementary Information).

The isolates were collected over 30 years, from 1986 to 2015. The study period was divided into three parts on the basis of the vaccine type/s used for routine vaccination in our setting: (i) period 1 (1986–1997; *n* = 82 isolates) was defined by the single use of WCV; period 2 (1998–2003; *n* = 82 isolates) was the transition period as WCV was used only for primary vaccination whereas ACV was used in boosters; and period 3 (2007–2015, *n* = 175 isolates), when ACV completely replaced WCV. Additionally, 10 previously characterized strains (B3313, B1900, B1706, B1917, B2726, B0366, B0549, B0610, B1916 and B3230) were included in the present study for comparison in the clonal relatedness studies. These strains have been described as being the most representative clones (PFGE profiles and MLVA types) circulating in several European countries during the period 1998–2015 [16–18].

### Pulsed-field gel electrophoresis (PFGE)

The genetic relatedness of all the *B. pertussis* isolates included in the present study was determined using DNA fingerprint by PFGE, which was performed as described previously [19]. The DNA fingerprint profiles were analysed with the GelCompare II v.4.6 software (Applied Maths) using the arithmetic UPGMA as a group method with a band tolerance of 1% and an optimization setting of 1%. Underrepresented PFGE profiles (*n * < 5 isolates) were grouped as “others”.

### Multi-locus variable-number tandem repeat analysis (MLVA)

Strain relatedness was also studied using MLVA typing analysis as described previously [20]. A subset of representative strains belonging to different PFGE profiles and time periods was selected for our MLVA comparison (*n* = 72). Accordingly, one isolate was selected from each of the less representative PFGE profiles (29 PFGE profiles, 29 isolates). For the most prevalent PFGE profiles (9 PFGE profiles, 43 isolates), 3 isolates were selected from each time period (1 from the beginning, one from the middle and 1 from the end of each period). In the case of PFGE profiles partially represented in a time period, available isolates were selected. Each locus was amplified by PCR and the resulting fragments analysed as described previously [20]. MLVA types (MT) were assigned using the MLVA typing tool (https://www.mlva.net/bpertussis/default.asp).

### Antigenic variants

A total of 231 strains belonging to different PFGE profiles were included in the characterization of antigenic variants: a subset of representative strains (*n* = 56) was selected for periods 1 and 2 whereas all strains collected during period 3 were analysed (*n* = 175). Among the genes encoding virulence factors included in the ACV, the A subunit of the pertussis toxin (*ptxA*), pertactin (*prn*) and type 3 fimbriae (*fim3*) were studied using PCR-based sequencing as described previously [21,22]. Additionally, the promoter type of pertussis toxin (*ptxP*) was also studied [14].

### Fimbrial serotyping

Fimbriae 2 (FIM2) and 3 (FIM3) serotyping of all 339 *B. pertussis* isolates included in the present study were performed by the indirect ELISA method as described previously [23,24]. Monoclonal antibodies (06/124; FIM2, and 06/128; FIM3) were obtained from the National Institute for Biological Standards and Control (NIBSC) and the reference strains Tohama I (FIM2), B1900 (FIM3) and B3313 (FIM2 and FIM3) were used as positive controls.

### Statistical analysis

PFGE profiles and MLVA types diversity were calculated based on the ratio between the PFGE profiles or MLVA types identified by the total number of isolates. The Simpson diversity index (SDI) was calculated based on the formula SDI = 1 − Σ*n*(*n* − 1)/*N*(*N* − 1) where *n* indicates the number of individual PFGE profiles or MLVA types identified and *N* indicates the number of all PFGE profiles or MLVA types observed [18].

## Results

### Study periods

All the isolates recovered in the present study (*n* = 339) were grouped into three study periods taking into account the gradual WCV replacement: period 1 (1986–1997, *n* = 82) was defined as WCV administration alone; period 2 (1998–2003, *n* = 82) was the vaccine transition period, and period 3 (2007–2015, *n* = 175) was characterized as exclusive ACV administration.

### Molecular typing: PFGE

The genetic relatedness of the 339 isolates was evaluated by PFGE. The results identified a total of 38 different PFGE profiles (Table S1, Supplementary Information). Of these, 19, 10 and 18 PFGE profiles from periods 1, 2 and 3, respectively were identified.

**Figure 1.**
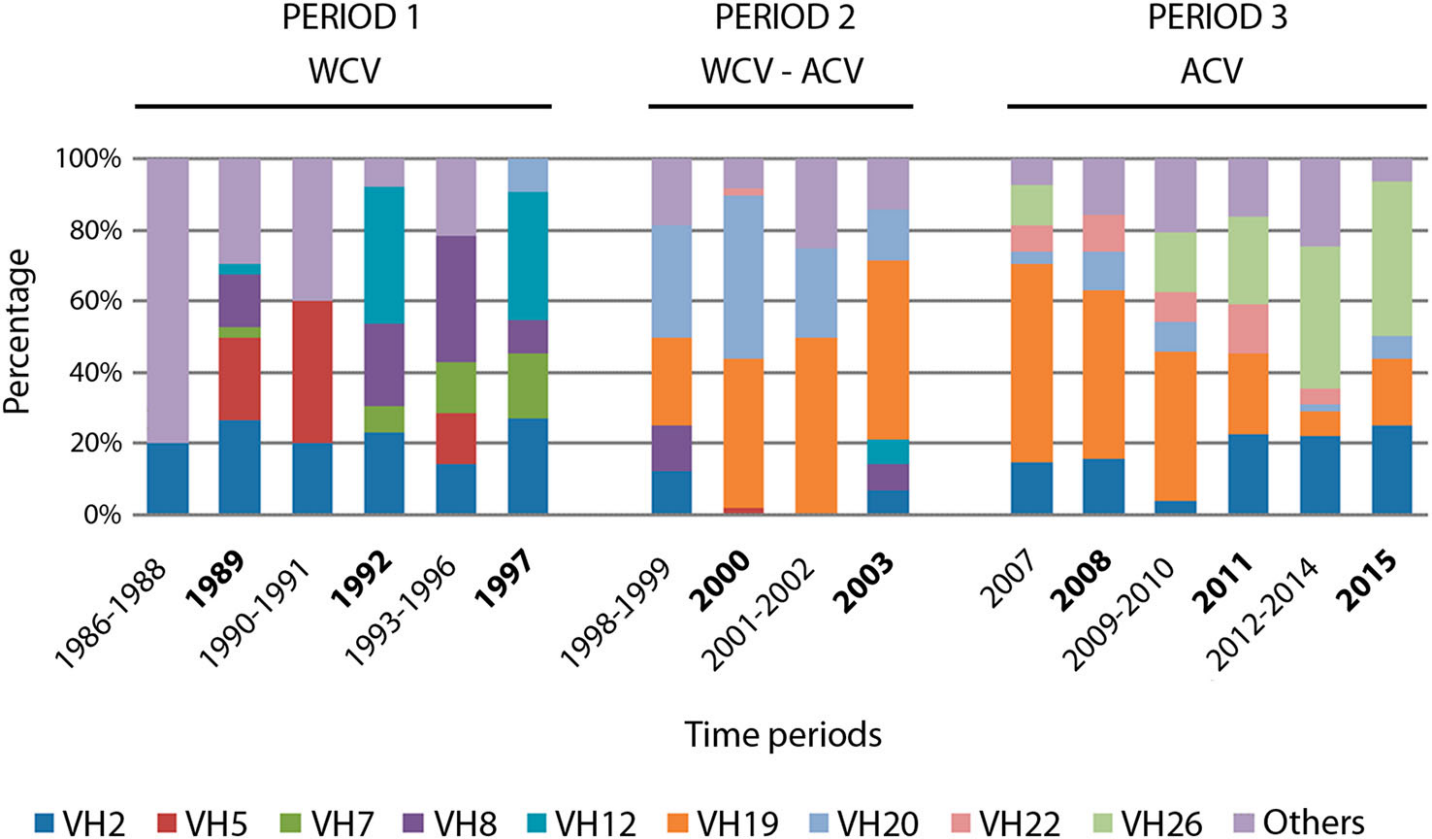
Temporal distribution of PFGE profiles of *B. pertussis* circulating in the metropolitan area of Barcelona from 1986 to 2015. WCV: Whole cell vaccine; ACV: Acellular vaccine; Bold-face type is used to indicate epidemic years.

VH2 was the only PFGE profile detected along the entire period (1986–2015), with a remarkable gap in the epidemic year 2000 and the following interepidemic years 2001–2002 (Figure 1). Additionally, a replacement of PFGE profiles was observed across the three periods, being more notable from period 1 to period 2. In period 1 (1986–1997, WCV) the PFGE profiles most frequently detected were VH2 (23.2%), VH8 (17.1%), VH5 (14.6%), VH12 (12.2%) and VH7 (7.3%). The introduction of ACV during period 2 (1998–2003) correlated with the emergence of VH19 (40.2%) and VH20 (36.6%), which were only marginally detected (1.2%) during period 1. Finally, in period 3 (2007–2015, ACV) PFGE profiles VH19 (28.6%), VH26 (24.6%), VH2 (18.3%) and VH22 (8%) were the most prevalent. During this last period, VH19 was the most frequent PFGE profile before the epidemic year 2011 (from 2007 to 2010, 48.6%; from 2011 to 2015, 15.2%), whereas progressive replacement by PFGE profiles VH26 and VH2 was observed from 2011 onwards (from 2007 to 2010, 10% and 11.4%, respectively; from 2011 to 2015, 34.3% and 22.9%, respectively) (Figure 1).

The PFGE profiles of clinical isolates obtained from several European countries from 1998 to 2015 were compared [16–18]. The results showed that the predominant European PFGE profiles BpSR3, BpSR5, BpSR10, BpSR11 and BpSR12 were respectively indistinguishable from VH26, VH22, VH2, VH19 and VH20 reported in this study, (Figure S2, Supplementary Information). General trends were similarly observed in both geographical areas (Europe and Spain) (Table 2): (i) BpSR11 (VH19) was the predominant profile during the period 1998–2005 (27–40%) and showed decreased prevalence in recent years (15–29%); (ii) BpSR10 (VH2) was less prevalent during the period 1998–2005 but has increased since 2006 (from 8% to 27% in Europe, from 4% to 18% in Spain); (iii) BpSR5 (VH22) has shown a similar low prevalence in both studies since its first detection in 1998 (1–11%); and (iv) BpSR3 (VH26) remained undetected from 1986 to 2003 but rapidly became one of the most prevalent types during the period 2007–2015 (22–29% in both areas). On the contrary, the greatest difference was seen for the PFGE profile BpSR12 (VH20). It was one of the most prevalent types in Spain (37%) from 1998 to 2003, thereafter it showed a remarkable decrease (4%) and was only a minor profile (4–7%) in Europe throughout the study period.

**Table 2.** PFGE profiles of *B. pertussis* circulating in the metropolitan area of Barcelona from 1986 to 2015 compared to PFGE profiles circulating in European countries from 1998 to 2015.

HVH PFGE profile	Time periods			European Equivalent PFGE profile^a^	Time periods			
	1986–1997 *n* = 82	1998–2003 *n* = 82	2007–2015 *n* = 175		EUpert I (1998–2001) *n* = 102	EUpert II (2004–2005) *n* = 154	EUpert III (2007–2009) *n* = 140	EUpert IV (2012–2015) *n* = 265
VH2	23	4	18	BpSR10	8	10	21	27
VH19	0	40	29	BpSR11	27	30	13	15
VH20	1	37	4	BpSR12	4	7	4	4
VH22	0	1	8	BpSR5	6	8	11	5
VH26	0	0	25	BpSR3	0	8	22	29
Total	24	82	84	Total	45	63	71	80

Note: Values are %.

^a^These data have been collected from EUpert studies [16–18].

### Molecular typing: MLVA

**Figure 2.**
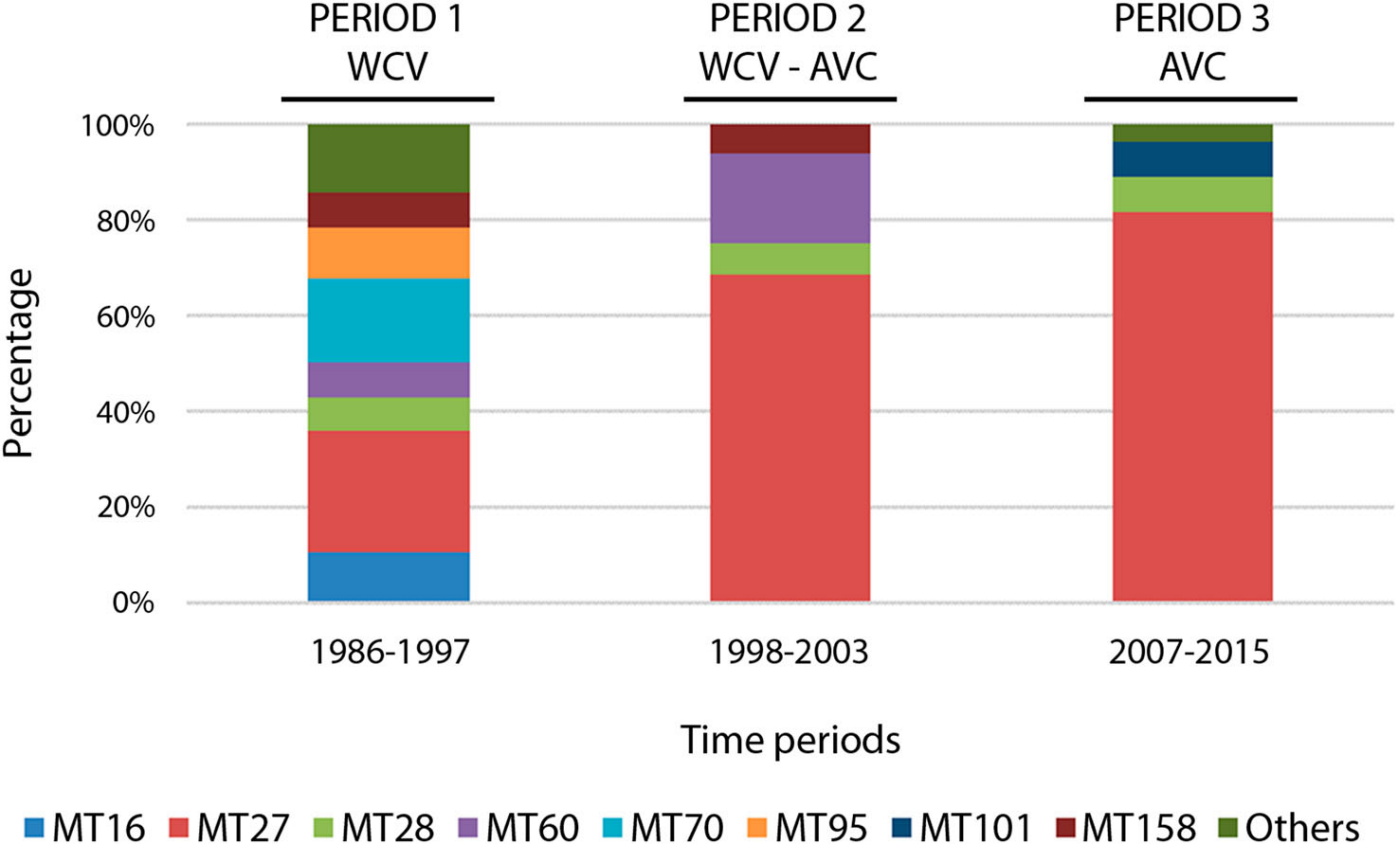
MLVA types of *B. pertussis* circulating in the metropolitan area of Barcelona from 1986 to 2015. WCV: Whole cell vaccine; ACV: Acellular vaccine.

MLVA typing was performed in 72 isolates selected as being representative of each PFGE profile (28, 16 and 28 isolates from each period, respectively) (Table S1, Supplementary Information). Overall, 13 different MLVA types (MTs) were found: 11, 4 and 4 MTs were detected during periods 1, 2 and 3, respectively. Among these, MT27 was the most prevalent type (56.9%) and clearly showed an increasing frequency: 25% during period 1, 68.8% during period 2 and 79.3% in the third period (Figure 2). Less representative MTs were MT16, MT28, MT60, MT70, MT95, MT101 and MT158, which were detected in more than one isolate with a prevalence per period ranging from 6.3% to 18.8%. Among these, MT28 was the only MT detected throughout the three periods (7.1%, 6.3% and 7.1%, respectively). The remaining MTs were exclusive of period 1 (MT70, MT16 and MT95), exclusive of period 3 (MT101) or present in both periods 1 and 2 (MT60 and MT158). Finally, five MTs were represented by single isolates: MT32, MT133, MT135 and MT146 from period 1 and MT30 from period 3 (3.6% individual prevalence per period).

### Comparative genetic diversity

The comparison of the genetic diversity among the three study periods based on differences in percentages of PFGE profiles and MTs showed a gradual decrease over time (Table 3). Thus, the diversity of the PFGE profiles identified decreased from 23.2% in period 1, to 12.2% in period 2, and to 10.3% in period 3. A similar and more evident decrease was seen for the MLVA-types, from 39.3% in period 1, to 25% in period 2, and to 14.3% in period 3. The Simpson’s diversity indexes calculated for each study period showed no differences in the transition from WCV to ACV on analysing the PFGE profiles (0.88 period 1, 0.70 period 2 and 0.82 period 3), whereas differences were noted when MLVA was taken into consideration (0.89 period 1, 0.52 period 2, and 0.33 period 3).

**Table 3.** Percentage of PFGE profiles and MLVA types identified and Simpson diversity index calculated for PFGE and MLVA in the three study periods.

Method	Genetic diversity analysis	Time periods		
		1986–1997	1998–2003	2007–2015
PFGE	Diversity (%)	23.2	12.2	10.3
	Simpson diversity index	0.88	0.70	0.82
MLVA	Diversity (%)	39.3	25	14.3
	Simpson diversity index	0.89	0.52	0.33

### ACV impact on the evolution of antigen variants and fimbrial serotype

**Figure 3.**
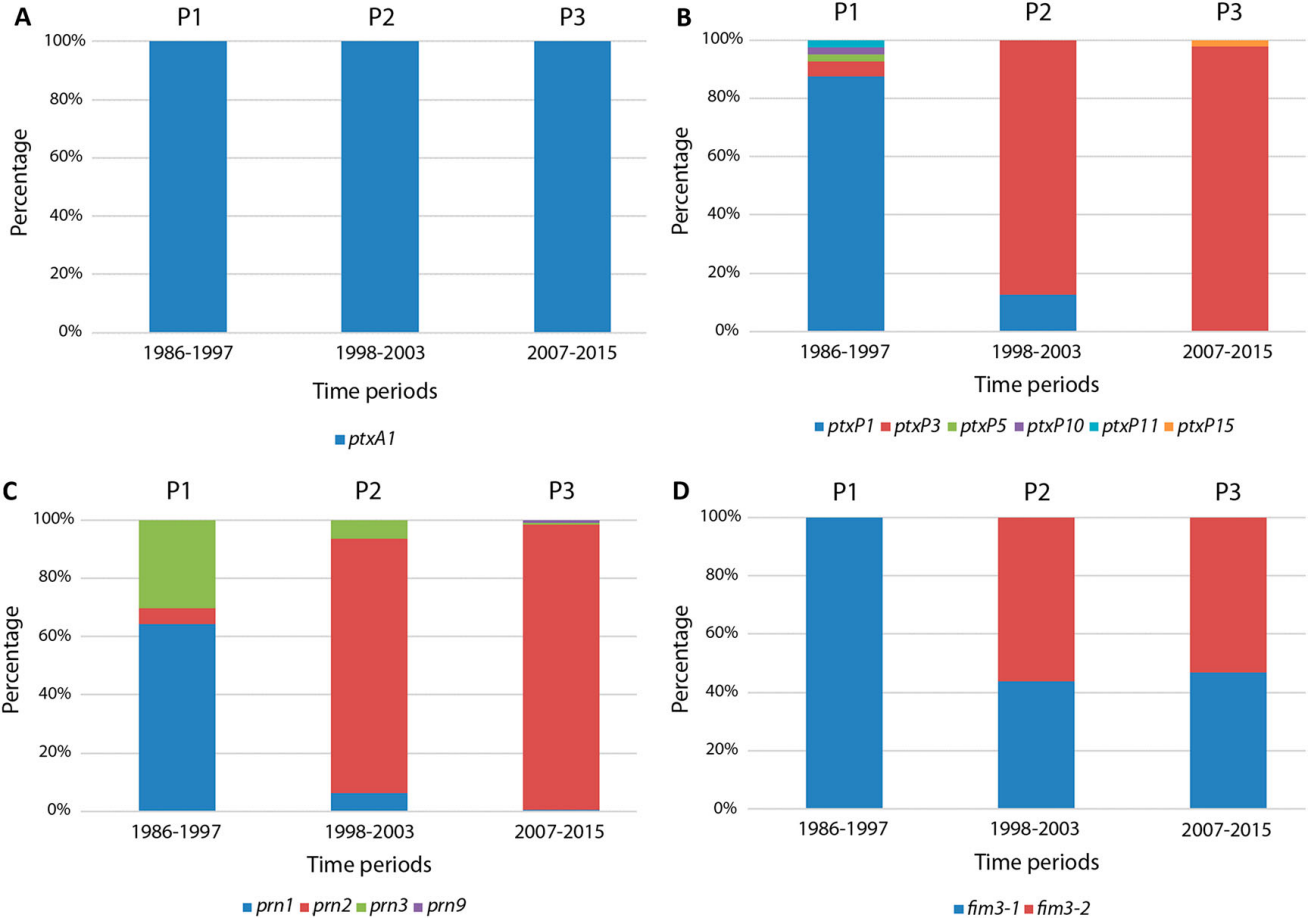
Antigen variants detected in *B. pertussis* circulating in the metropolitan area of Barcelona from 1986 to 2015. (A) Pertussis toxin. (B) Pertussis toxin promoter type. (C) Pertactin. (D) Type 3 fimbriae. P1: exclusive use of whole cell vaccine; P2: transition from whole cell vaccine to acellular vaccine; P3: exclusive use of acellular vaccine.

Allelic variant analysis was carried out in a selection of isolates to assess the prevalence and evolution of the most frequently studied vaccine components (PT, PRN and FIM3) among the *B. pertussis* population from Barcelona over the last 30 years (Figure 3). The promoter type of the pertussis toxin (*ptxP*) was also studied. A total of 231 isolates were analysed (40 belonging to period 1, 16 to period 2, and 175 to period 3).

Regarding the *ptxA* gene, our results revealed that all the isolates analysed carried the *ptxA1* allele (Figure 3(A)). In contrast, an important shift was observed concerning the promoter type. *PtxP1* was the most prevalent type while WCV was administered (period 1, 87.5%; period 2, 12.5%) but became undetectable when ACV was the only type of vaccine used in Spain (period 3) (Figure 3(B)). An inverse correlation was seen for the new *ptxP3* variant associated with higher PT production levels. It was first detected in 1996 (5%, period 1) and rapidly became the predominant allele (87.5% and 97.7% for periods 2 and 3, respectively). This predominance was seen in all the PFGE profiles analysed with the exception of VH5, a PFGE profile almost exclusive of period 1, as the only VH5 isolate collected in 2000 and analysed here harboured the *ptxP1* variant. Other less prevalent promoter types (2.3–2.5%) were observed in period 1 (*ptxP5*, *ptxP10* and *ptxP11*) and period 3 (*ptxP15*).

The pertactin gene allele distribution proved to be heterogeneous during period 1. Specifically, *prn1* and *prn3* were the most frequently detected (42.5% and 20%, respectively), while a lower frequency of *prn2* was detected (3.7%). Nonetheless, the *prn2* allele became the most predominant after the introduction of ACV, reaching a prevalence of 87.5% in period 2 and 97.7% in period 3 (Figure 3(C)).

**Figure 4.**
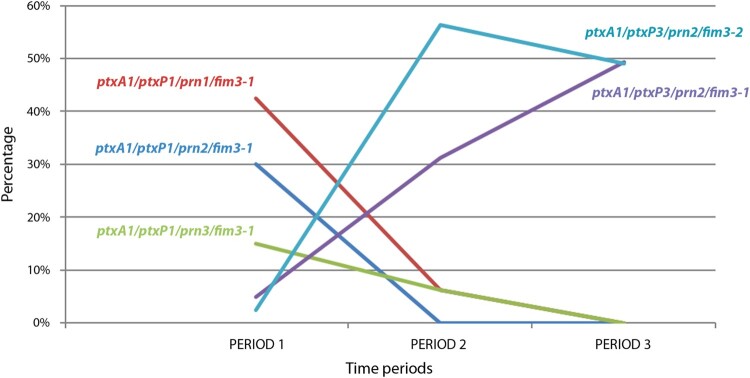
Temporal evolution of the most prevalent antigenic profiles observed in *B. pertussis* circulating in the metropolitan area of Barcelona from 1986 to 2015. Period 1: exclusive use of whole cell vaccine; Period 2: transition from whole cell vaccine to acellular vaccine; Period 3: exclusive use of acellular vaccine.

Finally, regarding the *fim3* genotype, our results showed that all the isolates studied in period 1 carried the *fim3-1* variant, whereas a similar prevalence of *fim3-1* and *fim3-2* variants were detected in periods 2 (43.8% and 56.3%, respectively) and 3 (46.9% and 53.1%, respectively) (Figure 3(D)). Intriguingly, from 2011 onwards *fim3-1* showed a progressive reemergence to again become the most predominant variant over *fim3-2* (data not shown). Thus, the 2 most prevalent allelic combinations detected in the present study during the most recent years were defined by isolates carrying the same allelic composition for *ptxA1*, *ptxP3* and *prn2*, while only differing in the allele encoding FIM3 (Figure 4).

**Figure 5.**
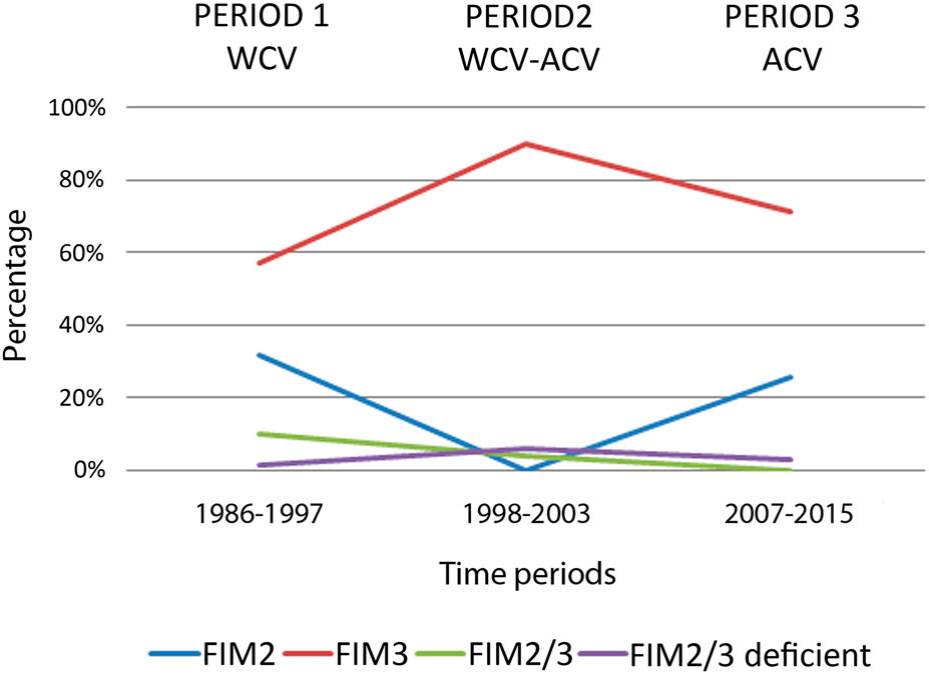
Evolution of fimbrial serotypes in *B. pertussis* isolates circulating in the metropolitan area of Barcelona from 1986 to 2015. WCV: Whole cell vaccine; ACV: Acellular vaccine.

Regarding fimbrial serotyping, overall 72.6% of the isolates had FIM3, 20.9% FIM2 and 3.2% FIM2/3. Additionally, 11 isolates (3.2%) were deficient for FIM2 and FIM3. The distribution of the serotypes per periods revealed that there was a significant increase of FIM3 isolates during the transition from WCV to ACV (57.3% during period 1,90.2% during period 2; *P < .05*) while FIM2 decreased dramatically (31.7% during period 1, 0% during period 2; *P* < .05). However, once the ACV was completely introduced (period 3), a progressive decrease of FIM3 isolates was detected (71.4%) as long as FIM2 isolates gradually reemerged (25.7%) (Figure 5). During period 1 (WCV), all FIM2 and FIM3 isolates were associated with a *fim3-1* genotype, while during period 2 (transition from WCV to ACV) 46.2% and 53.8% of the FIM3 isolates studied belonged to *fim3-1* and *fim3-2* genotypes, respectively. Finally, in period 3 (ACV), all the FIM2 isolates had the *fim3-1* genotype while both genotypes were identified (71.2% *fim3-2* and 28.8% *fim3-1*) for FIM3 isolates. There was no correlation among serotypes and vaccinated, non-vaccinated and partially vaccinated patients (*P > *.05).

Combining the analysis of PFGE profiles and fimbrial serotype, most of the FIM2 isolates belonged to PFGE profiles VH26 and VH2 (42.3% and 18.3%, respectively) while most of the FIM3 isolates belonged to PFGE profiles VH19, VH2 and VH20 (32.1%, 15.4% and 14.2%, respectively). Regarding the most common PFGE profiles, isolates belonging to VH2 and VH26 included positive isolates for the FIM2 (24.1% and 69.8%, respectively) and also the FIM3 (70.4% and 27.9%, respectively) serotypes, whereas PFGE profiles VH19, VH20 and VH22 were almost exclusively FIM3 (95.2%, 92.1% and 100%, respectively). There was no significant association between FIM2/3, or FIM2 and FIM3 deficient isolates and specific PFGE profiles (Table S1, Supplementary Information).

## Discussion

Changes in the *B. pertussis* population have been reported worldwide [2–4,13]. Nonetheless, there are no data related to Spanish strains. In this study, 339 *B. pertussis* isolates collected in Barcelona during the period 1986–2015 were analysed to assess the molecular evolution of this pathogen. Moreover, the global impact of the vaccine transition strategy from WCV to ACV was assessed by integrating data from molecular typing and antigenic variance. The results of this study show a clear shift in the *B. pertussis* population as well as in its antigenic profile concurrent with the introduction of ACV, as noted in other countries.

Only isolates showing the VH2 PFGE profile were found to persist throughout the entire study period. Isolates belonging to PFGE profiles VH8, VH5 and VH12, which were predominant during the years in which WCV was the only vaccine administered, were almost completely replaced by the newly appearing profiles VH19, VH20 and VH22, which became dominant once ACV remained as the only vaccine in use. Finally, PFGE profile VH26 appeared in 2007 and has become the most prevalent profile since 2011. These results are largely consistent with the most prevalent PFGE profiles detected in Europe within the same 30-year time frame [2,16–18]. Our findings are also in agreement with the predominant PFGE profiles circulating in the US since 2000 [3]. Overall, these results reinforce the successful intercountry dissemination of *B. pertussis* lineages not only among neighbouring countries but also among overseas territories with similar vaccination policies. However, slight variations in the prevalence of the distribution of some PFGE profiles were noted on comparing local and European isolates. Specifically, the PFGE profile VH20 was one of the most prevalent types in Barcelona from 1998 to 2003 (37%) but showed a noteworthy decrease from 2007 to 2015 (4%). On the contrary, its equivalent European PFGE profile (BpSR12) was only a minor profile in Europe during the whole study period (4–7%). This fact highlights the need for regional studies to fully understand population dynamics within and across territories. Probably, the introduction of whole-genome sequence in future studies may shed new light on the understanding of the evolution and rate of lineage mixing within areas.

Next, we assessed whether there are epidemiological differences between epidemic and inter-epidemic periods. The percentage of minority PFGE profiles was clearly increased in the latter, particularly for period 1 and less pronounced in period 2. It is of interest that the VH12 PFGE profile was only detected during the epidemic years 1989, 1992 and 1997. These results suggest that some epidemic waves might be related to particular clones sharing specific characteristics, what could contribute to increased dissemination potential.

The MLVA results supported the PFGE findings regarding temporal trends and dynamic changes in the population structure of *B. pertussis* in Barcelona over time. A trend to a lower diversity of MTs was observed in concurrence with the switch in vaccines, mainly due to the growing dominance of MT27 (25% during WCV administration from 1986 to 1997, to 79.3% once ACV was completely implemented). Similar figures have been reported across Europe as well as in the US and Australia since the introduction of ACV [2,13,18,25]. In contrast, the prevalence of MT27 is marginal in countries in which WCV is still used, or has been used until only recently, such as in China (6% in period 2012–2013), Poland (2.9% in period 1959–2013) or the Philippines (not detected) [26–28].

Apart from changes in the bacterial epidemiological profiles, we also assessed the impact of the introduction of ACV on the allelic selection of vaccine-related virulence determinants. We found that all the isolates collected across the entire study period were *ptxA1*, in agreement with previous reports in which *ptxA1* was described as being the most prevalent variant at the end of the 1960s immediately after the introduction of the WCV [9]. Consequently, as the PT variants included in the ACV are encoded by *ptxA2* and *ptxA4* alleles, a protective effect against *ptxA1* is no longer expected.

On the contrary, two remarkable antigenic shifts were found: the first was related to the new PT promoter variant *ptxP3*, and the second to the *prn2* allele. Strains carrying the *ptxP3* variant have been characterized by increased toxin production levels and higher virulence potential [14]. Moreover, such strains have been associated with the reemergence of the pathogen in most parts of the world since the 1990s, concomitant to a shift towards older age groups [13,14,29]. The abrupt replacement of *ptxP1* by *ptxP3* seen in period 2 was paralleled by a similar change in the prevalence of PRN variants, with *prn2* becoming dominant after almost completely displacing *prn1*. This is in the line of previous reports showing a similar shift towards a clear dominance of the *prn2* allele after the introduction of ACV, which includes the allelic variants *prn1* and *prn7* which were most frequently detected before the withdrawal of WCV [9,18].

Lastly, we also completed the antigenic profile of our isolates by the characterization of the FIM3 genotype, which is absent in some commercially available ACVs. To date, two alleles have been reported as being the most prevalent: *fim3-1* and *fim3-2*. The results of our study show that all the strains analysed from period 1 only carried the *fim3-1* variant, whereas a substantial decrease was subsequently observed towards a similar proportion (43.8% for period 2 and 46.9% for period 3). Nonetheless, since 2011 *fim3-1* has shown a progressive reemergence towards the predominant variant. Similar results have been reported in Dutch isolates which showed a transitory increased prevalence of *fim3-2* strains which reverted a few years later [30]. Although overall most of the isolates were FIM3 (72.6%), on stratifying the results according to the vaccine administered once ACV was introduced, a progressive shift from FIM3 to FIM2 was observed, even though most of ACV compositions used in our area do not contain any fimbrial antigen (Table 1). Similar results were reported in different European countries, such as Denmark and Finland, where ACV does not contain fimbrial components and FIM2 isolates were the most prevalent from 2012 to 2015. Conversely, FIM3 isolates were predominant during the period 2012–2015 in France, United Kingdom and Sweden, although the ACV contained fimbrial components only in the first two [18]. Therefore, factors other than the type of fimbrial antigen contained in the vaccine may be involved in the serotype shift. On the other hand, comparing the association of fimbrial serotype with PFGE profiles we observed that, as previously found in the EUpert III and IV studies, isolates belonging to VH19 (BpSR11), VH20 (BpSR12) and VH22 (BpSR5) PFGE profiles were mostly associated with FIM3 regardless of the period in which they were isolated. However, during the transition from WCV to ACV, all VH2 (BpSR10) and VH26 (BpSR3) isolates were FIM3 while a shift towards the FIM2 serotype was observed once the ACV was fully implemented [17,18].

According to the results of the present study, the alleles detected in the most prevalent isolates circulating in our population since 2011, and at least until 2015, were as follows: *ptxA1* for the pertussis toxin, which is regulated by a *pxtP3* type promoter, *prn2* for pertactin and *fim3-1* for serotype 3 fimbriae. The corresponding shifts in the antigenic profile of the strains can be partially correlated with PFGE profile replacement, particularly in period 3: the PFGE profile VH19 (mainly *ptxA1*, *ptxP3*, *prn2*, *fim3*-*2*) has been replaced by VH26 (mainly *ptxA1*, *ptxP3*, *prn2*, *fim3-1*). These results suggest that the absence of uniformity in vaccine composition has failed to counteract the dissemination of *fim3-1* isolates. Dissemination of VH26 could be supported by a bacterial advantage provided by this fimbrial allele or by another virulence trait specific of this clone. Conversely, VH2 (mainly *ptxA1*, *prn2*, *fim3-1*) was detected throughout three study periods, and several factors may have influenced its continuous detection: no selective pressure on pertactin together with an inner adaptation ability, highlighting that the same PFGE profile acquired the widely disseminated *ptxP3* variant. Thus, the adaptative plasticity of *B. pertussis* facilitates the diversity and selection of the most appropriate clones in addition to an evolving ability to integrate chromosomal changes in the same clone.

In conclusion, these results support the presence of dynamic changes in the *B. pertussis* population as well as in its antigenic profile in Barcelona (Spain) over a 30-year time period, with a trend towards homogenization following the introduction of the ACV. Our findings suggest that the use of the ACV has likely driven the population changes and antigenic shift of *B. pertussis*.

## Supplementary Material

Supplemental Material
