# Supplementary figures and images for: Population dynamics and antigenic drift of Bordetella pertussis following whole cell vaccine replacement, Barcelona, Spain, 1986–2015
Source: Emerg Microbes Infect. 2019 Nov 26;8(1):1711–20. doi: 10.1080/22221751.2019.1694395 (PMC6882445; doi:10.1080/22221751.2019.1694395)

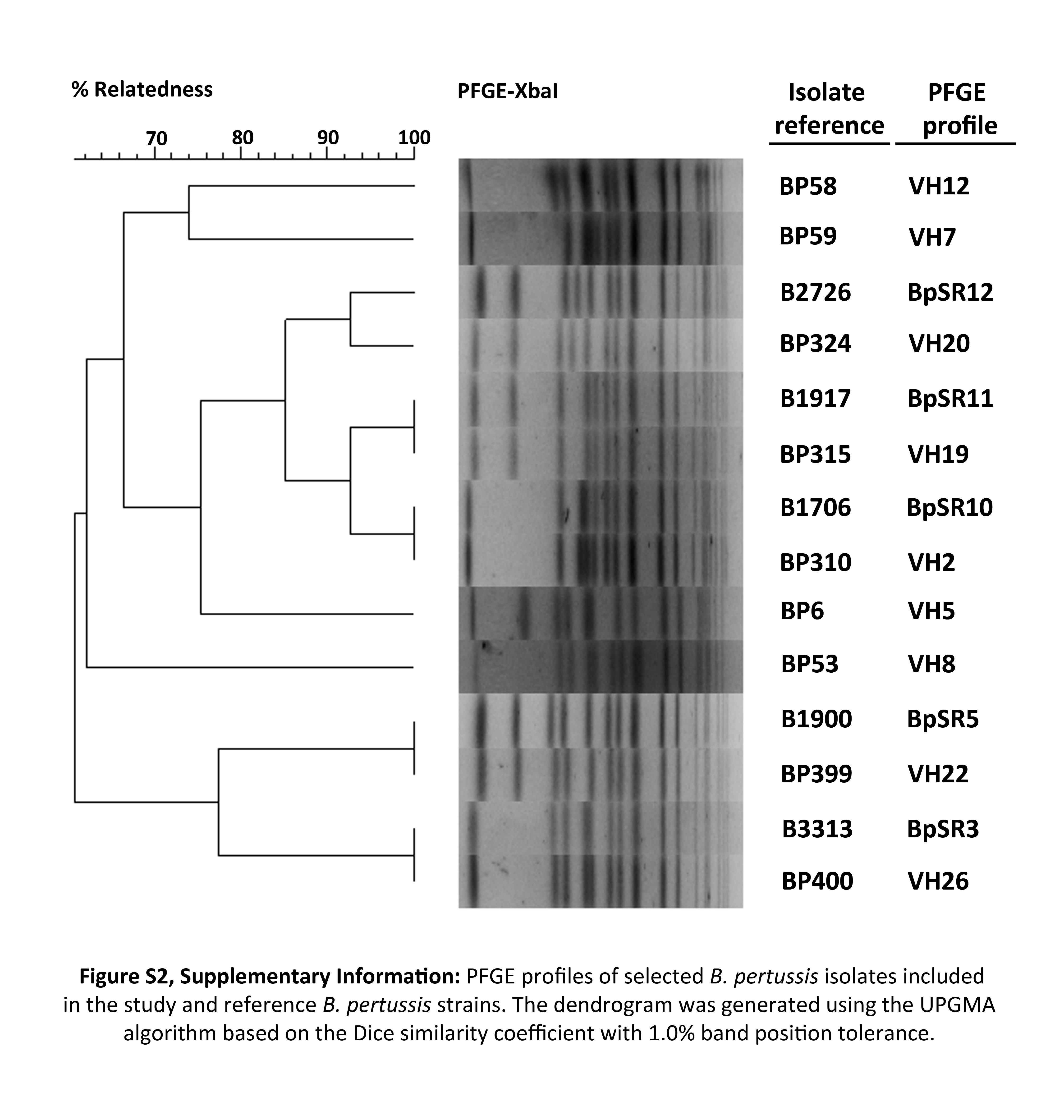

Supplement: Supplemental Material [file TEMI_A_1694395_SM0707.zip › Figure S2, Supplementary Information_final.tif]
